# A Bibliometric Analysis (2010–2020) of the Dental Scientific Literature on Chemo-Mechanical Methods of Caries Removal Using Carisolv and BRIX3000

**DOI:** 10.3390/medicina58060788

**Published:** 2022-06-11

**Authors:** Dana Cristina Bratu, Nicoleta Nikolajevic-Stoican, George Popa, Silvia Izabella Pop, Bianca Dragoș, Magda-Mihaela Luca

**Affiliations:** 1 Department of Orthodontics and Dento-Facial Orthopedics, Orthodontic Research Center, Faculty of Dental Medicine, “Victor Babes” University of Medicine and Pharmacy Timisoara, 2 Eftimie Murgu Square, 300041 Timisoara, Romania; bratu.cristina@umft.ro; 2 Department of Pediatric Dentistry, Pediatric Dentistry Research Center, Faculty of Dental Medicine, “Victor Babes” University of Medicine and Pharmacy, 2 Eftimie Murgu Square, 300041 Timisoara, Romania; nicoleta.stoican@umft.ro (N.N.-S.); luca.magda@umft.ro (M.-M.L.); 3 Department of Orthodontics, Faculty of Dental Medicine, George Emil Palade University of Medicine, Pharmacy, Science, and Technology of Targu Mures, 38 Gheorghe Marinescu Street, 540142 Targu Mures, Romania; 4 Research Center in Dental Medicine Using Conventional and Alternative Technologies, Faculty of Dental Medicine, “Victor Babes” University of Medicine and Pharmacy, 9 Revolutiei 1989 Avenue, 300070 Timisoara, Romania; bianca.roman@umft.ro

**Keywords:** Carisolv, BRIX3000, authorship, citation, VOSviewer

## Abstract

Reports in the literature have proposed and analyzed several minimally invasive techniques for caries removal in recent decades. In light of recent events surrounding the ongoing epidemiological context, concerns have been raised regarding the generation of aerosols during dental procedures. The aim of our research was to provide an overview of the scientific literature on the topic of chemo-mechanical caries removal (CMCR) methods, focusing on two products (Carisolv, BRIX3000), commercially available in Europe. A bibliometric analysis was used to investigate the scientific articles included in Web of Science (WoS) Core Collection database, published from January 2010 to December 2020. We analyzed the co-occurrence of all keywords (Author Keywords and the KeyWords Plus section), co-authorship and co-citation, using the free software VOSviewer. Our bibliometric analysis revealed a worldwide interest in the subject of chemo-mechanical methods of caries removal, which has transcended the area of pediatric dentistry. The analyzed studies have been conducted mainly in high-income countries that have developed sanitary policies regarding prevention and early treatment of carious lesions as a health priority.

## 1. Introduction

The subject of chemo-mechanical removal of dental caries has gained the interest of the scientific world in recent years, in connection with the increased demand for minimally invasive methods in dentistry, including the atraumatic restorative technique (ART) that is predominantly used in pediatric patients. The topic, however, transcends the field of pediatric dentistry, with numerous articles being published in journals of different specialties, such as The Journal of Endodontics or The Journal of Prosthetic Dentistry.

The removal of dental caries without generating aerosols has been addressed for several decades, in an attempt to find an alternative treatment method for pediatric patients with dental anxiety [1,2].

The chemo-mechanical caries removal (CMCR) methods have gained increased acceptance among children because the procedures are simple, painless and overcome many of the drawbacks of conventional caries excavation using rotary instruments (e.g., pressure and heat generation—harmful for the vital dental pulp, noise, vibration, pain and the need for local anesthesia) [3,4,5]. CMCR, which is a minimally invasive treatment technique, reduces the production of aerosols by using only hand instruments in caries excavation, avoiding splash and splatter and coughing/gag reflex. The splatter and dissemination of contaminated aerosols that result from cavity preparation remain a serious threat to dental staff, especially during the Coronavirus Disease 2019 (COVID-19) pandemic [6].

Currently, there are several CMCR products available on the market, mainly used in pediatric dentistry, in primary teeth. Carisolv (RLS Global AB, Sweden) is a caries-removal agent first marketed in Sweden in 1998. The system consists of two solutions that are mixed together to form a gel containing sodium hypochlorite, sodium chloride, carboxy-methyl-cellulose and three amino acids (glutamic acid, leucine, and lysine); the gel selectively softens the demineralized carious dentine, while preserving healthy tissue [7]. The product does not cause any reaction in healthy dental pulp, which is one of the main advantages that makes this material suitable for maintaining pulp vitality in primary teeth [8].

BRIX3000 (Brix SRL, Argentina), a sodium hypochlorite-free agent, was first manufactured in Argentina and released internationally on the market in 2016. It is an enzymatic gel that induces the proteolysis of collagen from the affected dentin. Its active ingredient, papain (3.000 U/mg, 10%), is an endoprotein obtained from green papaya (Carica Papaya) that is similar to human pepsin and has bactericidal, bacteriostatic and anti-inflammatory activity [9]. Papain was bio-encapsulated with a neutral pH buffer using encapsulating buffer emulsion (EBE) technology [10].

Papacárie, a precursor of BRIX3000, was launched on the market in 2003. Later on, the formula for Papacárie was improved, and the gel-type product was made of papain, chloramine T, pectin, propylene glycol, polyethylene glycol and coloring agents [11].

According to some authors, BRIX3000 showed lower cytotoxicity [12,13] and a reduced removal time compared to Papacárie Duo [12].

The characteristics of the residual dental tissue after CMCR can influence the strength of adhesion between the restorative material and the dental substrate. Using Carisolv on extracted teeth, Peric and Markovic [14] observed under scanning electron microscopy (SEM) several differences in the morphology of the cavity floor: the dentinal surfaces of primary teeth treated with Carisolv were rough and irregular, with minimal smear layer and patent dentine tubules; the permanent teeth showed extremely irregular dentinal surfaces, minimal smear layer and patent dentine tubules.

The microtensile bond strength at the interface with the restorative material after the use of Carisolv on permanent molars did not suffer any modifications compared to the conventional technique using dental burs or other minimally invasive techniques, such as sonic preparation and air abrasion [15]. Similar results were found for both Papacárie and Carisolv in a different study by Hamama, Yiu and Burrow [16].

In a study on primary teeth, Cecchin et al. [17] found no significant differences in the microtensile bond strength of adhesives between the CMCR techniques using Carisolv and Papacárie. On the other hand, Pravin Maru, Shakuntala and Dharma [18] found that the temporary teeth treated with Papacárie showed less marginal leakage and higher shear bond strength when compared to conventionally treated teeth.

The use of CMCR methods overall improves the therapeutic protocols through simple-to-follow treatment steps, increased treatment acceptance and better patient cooperation, being as reliable as conventional techniques of caries removal and producing optimal tooth surface characteristics.

The study was based on articles published from January 2010 to December 2020, considering the growing interest in the last decade related to the CMCR methods. This ascending trend can only have a positive impact on the pediatric population, with multiple ramifications related to oral health: increased treatment acceptance from the earliest age, preservation of dental tissue, early treatment of temporary and young permanent teeth with emphasis on prevention and minimally invasive treatment, thus avoiding extractions and potential dental malpositions.

The association of ART with chemo-mechanical methods has been previously investigated in elderly patients [19], but it came into the spotlight in 2020, when the unique situation created by the COVID-19 pandemic emphasized the need for dental procedures that produced as few aerosols as possible [20]. Anxiety and fear also had an important impact on dental practitioners worldwide; some decided to limit their services to emergency treatment only or to close down their offices due to the pandemic [21]. In this context, we wanted to assess the general interest worldwide related to using CMCR methods as an aerosol-free treatment alternative.

Including the aspects we already mentioned and several other parameters we considered relevant, we conducted a bibliometric analysis of the dental scientific literature on CMCR methods, focusing on the use of Carisolv and BRIX3000. Bibliometric analysis is an approach for quantifying scientific publications in order to estimate a journal’s research output, using several descriptive statistics, such as citation data and network analysis, which cover journals, authors, nations, keywords, and academic and research organizations.

This study can be used as a reference point for future research to compare the evolution of implementing these minimally invasive procedures on a larger scale in the standard therapeutic protocols.

## 2. Materials and Methods

The analyzed data were collected from the Web of Science (WoS) Core Collection database, which is one of the most important sources in the world of interconnected research information that includes only high-quality academic journals.

The search was focused on two chemo-mechanical materials used for caries removal, commercially available in Europe (Carisolv, BRIX3000). For the search criteria, we selected the option “Topic” in the search engine menu of WoS Core Collection and used the expression “BRIX3000” OR “BRIX 3000” OR “Carisolv”. The initial sample included 68 publications from January 2010 to December 2020. The final data set was limited to 66 documents by selecting only the articles and review articles written in English and excluding documents written in other languages. We verified that the content of all of the articles was relevant to our analysis. The resulting data set was exported as a tab-delimited file with a txt file extension. The file was imported as a bibliographic database in VOSviewer (version 1.6.18). A thesaurus file was also used to merge all synonym keywords into a single term; for example, the word “Carisolv” was chosen to replace the following keywords: “Carisolv (r)”, “Carisolv (tm) gel”, “Carisolv gel”, “Carisolv III” and “Carisolv (tm)”. The last search through the WoS database was performed on 16 January 2022.

VOSviewer is a free software product that was used to create and visualize bibliometric networks and to provide information on research clusters, current interests and emerging subject trends. A bibliometric network contains scientific publications or journals, keywords, researchers, countries, research organizations or other inclusion criteria terms. A network can be built based on citation, scientific co-authorship, co-occurrence, co-citation and bibliographic links. According to VOSviewer Manual [22], a link is a relation or a connection between two items. Each link has a strength represented by different positive numerical values related to the type of analyzed items; for example, the number of documents two researchers have co-authored (in the case of co-authorship links), or the number of cited references two documents have in common (in the case of bibliographic coupling links) [22].

In our study, we focused on the following types of analysis: co-authorship, co-occurrence of all keywords (more precisely, keywords that appeared in the Author Keywords section and the KeyWords Plus section) and co-citation (two documents that received a citation from the same document).

## 3. Results and Discussion

### 3.1. Keyword Analysis

**Figure 1:**
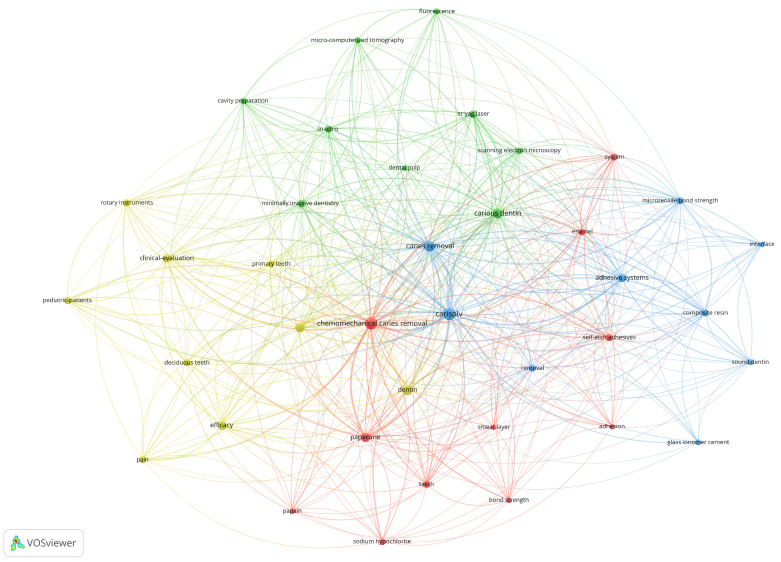
Map of the network of keywords for the CMCR methods (Carisolv and BRIX3000), based on the keyword co-occurrence. Source: own processing through VOSviewer.

This method highlights the most relevant keywords based on their occurrences, indicated by the number of articles in which a keyword occurs at least once. For our set of data, we chose to generate in VOSviewer a map of the network of keywords with a minimum of four occurrences in the analyzed articles, such as Carisolv (47 occurrences), chemo-mechanical caries removal (37 occurrences), caries removal (30 occurrences) and Papacárie (19 occurrences). The most important keywords and the links between them are shown in Figure 1: a larger node (keyword) indicates a greater weight (a higher number of occurrences); a smaller distance between the nodes indicates a stronger relationship between them; the same color indicates a series of related keywords or a group of keywords.

When the threshold was set at a minimum of four occurrences, the software generated a total of four clusters, which were further identified as Group 1 to 4. These groups and their related keywords are described in Table 1.

Group 1 (red) mainly focused on the effects of chemo-mechanical caries removal methods on the properties of the smear layer [16,23,24,25] and how these parameters influenced the adhesion [16,26,27,28,29,30] and the bond strength of dental adhesives [2,16,27,31,32,33,34,35]. Papacárie, a papain-based gel, was used in several studies to compare the efficacy of different CMCR methods [12,16,23,30,31,36,37,38,39,40,41,42,43,44,45,46,47]. Sodium hypochlorite was also tested in comparison with other CMCR agents, especially for endodontic use [26,46,48,49,50].

Group 1 also contained information about the principle of chemical alteration of the carious tissue, followed by its removal using hand instruments. The products used in this technique can be classified as either chlorine-based (sodium hypochlorite, NaOCl) or enzyme-based compounds [48]. The evolution of sodium hypochlorite agents was initiated by the use of GK 101, a 5% NaOCl solution. This formula was subsequently improved with sodium hydroxide, sodium chloride, and glycine to overcome the unpleasant effect of sodium hypochlorite and was named Caridex [2,48]. Carisolv contains a 0.95% NaOCl solution mixed with three amino acids (leucine, lysine, and glutamic acid), a combination that has a non-specific proteolytic effect on organic tissue. It is thought that positively and negatively charged groups of amino acids become chlorinated and further disrupt the collagen cross-linkage in the matrix of carious dentin, selectively dissolving the caries-infected dentin [26].

The enzyme-based agents contain papain, which has a proteolytic effect and interacts with the exposed collagen, resulting in its deterioration. The gel can also be used to dissolve the minerals from dentin tissue, softening and facilitating the removal of infected dentin [48]. The absence of a smear layer after chemo-mechanical caries removal is most likely due to the unique technique of preparation without thermal effects, the high pH level of the gel, and the possibility of smear layer dissolution caused by sodium hypochlorite, the active component of Carisolv gel. Other studies revealed that exposing the dentin surface alone to the action of a sodium hypochlorite solution not only resulted in the opening of dental tubules, but also in the occlusion of the vast majority of them. The addition of amino acids to the Carisolv formula aids in the dissolution of the smear layer and, as a result, the opening of the dentin tubules [2,48]. The proper adaptation of the restorative material to the tooth structure is one of the most important conditions for adhesion [26]. It appears that dentin adhesion is influenced by the adhesive method employed, as well as the properties of the dentin substrate [2,48]. The use of self-etch adhesives was studied by Hamama et al. [16] on permanent molars, divided into 3 groups, where group 1 was treated with Papacárie, group 2 with Carisolv and group 3 with conventional rotary instruments; the authors found that the bond strength of the three caries excavation procedures did not differ significantly and that chemo-mechanical caries removal had no influence on the bonding strength of self-etching adhesives. In another study, following the use of Papacárie, the excavated dentine surface was rough and characterized by a total absence of the smear layer, with predominantly patent dentinal tubules [42]. On the other hand, the excavated surface after the use of Carisolv was irregular and partially covered by a smear layer, with most of the dentinal tubules being patent or partially occluded [24,42]. After caries removal, the authors concluded that these morphological traits may improve the dentinal surface area available for micromechanical retention of the adhesive resin [16].

Group 2 (green) included the most relevant articles that studied the efficiency of removing carious dentin using minimally invasive dentistry [42,48,51,52], assisted by Er:YAG lasers [27,32,34,47,53,54] and fluorescence-aided caries excavation (FACE) [55,56,57]. The efficiency of these methods was assessed in several in vitro studies, using micro-computerized tomography [52,54,55,58,59,60] and scanning electron microscopy [24,45,49,53].

This group also established the relation between the CMCR treatment and the dental pulp response [24,51,61,62]. When choosing a restorative material, the dental specialist should take into consideration the use of biocompatible materials that are both well tolerated by the dental pulp tissue and may also offer other benefits, such as inducing dentin formation. According to the findings of Bussadori et al. both Papacárie and Carisolv proved to be non-cytotoxic for the pulp fibroblasts, maintaining the production of fibronectin and type I collagen and stimulating the production of osteonectin [61]. Because osteonectin is a calcium-binding protein, the presence of osteonectin and type I collagen in dental pulp fibroblasts implies that these cells have the ability to participate in the mineralization process during dentinogenesis [61]. In order to maintain the principles of minimally invasive dentistry, Zhang X. et al. [55] suggested that fluorescence-aided caries excavation (FACE) can be more effective at removing carious dentine, obtaining a cleaner surface than the conventional excavation, with less bacterially infected dentine. In an attempt to surpass the efficiency of the CMCR methods, the use of Er:YAG laser was analyzed by Neves, Coutinho, De Munck and Van Meerbeek in combination with laser-induced fluorescence (LIF), but the results showed a low efficacy in caries removal and low minimally invasive potential compared to chemo-mechanical excavation; thus, its use could not be regarded as a method for selective caries removal [54].

Group 3 (blue) focused on different aspects related to chemo-mechanical caries removal methods, especially those that involved the use of Carisolv [2,4,5,16,24,30,31,33,34,35,36,41,42,43,46,49,50,51,54,55,57,59,60,63,64,65,66,67,68,69]. Similarly to the first group, the articles included in this group also studied different adhesive systems and the microtensile bond strength or the microleakage of the interface between the residual dentin and composite resin [27,32,33,34,35,36,47] or glass ionomer cement [19,36,64,68].

According to some researchers, conventional glass ionomer cement showed higher values of microtensile bond strength for the dentine surfaces chemo-mechanically treated with Carisolv than those treated with Papacárie [36]. Carisolv did not negatively affect the microtensile bond strength of either the resin-modified glass ionomer cement or the conventional glass ionomer cement [36]. Similar results were reported by Mayaho et al., who studied the surface roughness of the dentin from forty temporary molars treated with four different minimally invasive methods using scanning electron microscopy (SEM), atomic force microscopy (AFM) and profilometry; the authors observed that the surface topography of the treated dentin revealed distinctive patterns according to the particular method used, but the shear bond strength of the glass ionomer cement was not noticeably altered by the use of any minimally invasive procedure [26].

Zawaideh, Palamara and Messer investigated the adhesion of composite resin to a dentine substrate treated with Carisolv on 45 extracted primary molars, concluding that the CMCR treatment did not affect the bond strength of resin composite materials [62].

Group 4 (yellow) focused on the clinical aspects of using CMCR methods on deciduous teeth [4,5,35,38,42,45,46,54,57,59,69,70], mainly on the pain management in pediatric patients [4,5,38,44,48,66,70,71,72] and on the efficacy of CMCR methods when compared to rotary instruments [4,37,47,54,63,66].

The CMCR methods are gaining ground because they avoid unnecessary removal of sound tooth structure, they minimize or eliminate the use of local anesthesia, are economical, and they require minimal armamentarium compared to all other advanced methods [73]. Despite requiring a longer time to remove carious tissue, Carisolv showed substantially higher performance in terms of anesthetic requirement, discomfort experienced by patients, and preference over conventional treatments; all of these findings promote Carisolv as a gentle and non-traumatic approach for caries removal, particularly in pediatric patients [74]. One study evaluated the pain and anxiety generated by different treatment techniques, using the Wong-Baker Faces scale, and concluded that BRIX3000 and 2.25% sodium hypochlorite gel Carisolv performed as a well-tolerated method of removing infected dentine in deciduous and permanent teeth [48]. Based on the available evidence, the currently used chemo-mechanical caries removal procedures can be regarded as a feasible alternative to conventional caries removal methods. These techniques might be particularly beneficial to patients who are severely anxious, disabled, or young children [2], but the extended excavation time required when using Carisolv gel should be taken into account when choosing the best caries removal approach for each clinical case.

### 3.2. Analysis of “Co-Authorship” in Terms of Number of Documents, Universities and Countries

**Figure 2:**
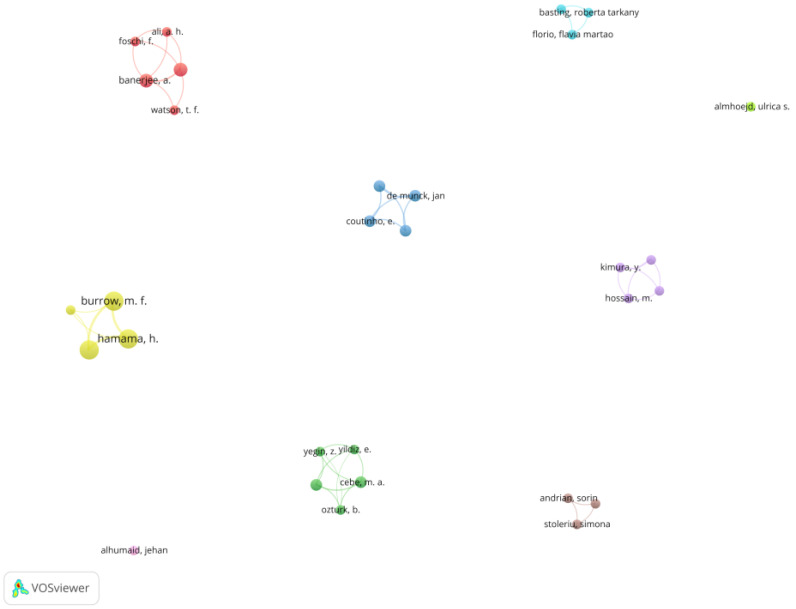
Network of scientific co-authorship, based on the number of documents per author. Source: own processing through VOSviewer.

In this section, the analysis focused on the research area of the main authors’ network. Concentrating on the two targets of analysis (Carisolv and BRIX3000), out of the 264 authors, 33 authors met the threshold of a minimum of two published documents per author, 11 authors met the threshold of a minimum of three published documents per author, four authors met the threshold of a minimum of five published documents per author and three authors met the threshold of seven published documents per author. Figure 2 shows the network of authors organized into 10 groups, according to the weight of the documents. The author groups are further detailed in Table 2.

Considering the relative novelty of these CMCR products (especially BRIX3000), the number of published articles was not high, but they made a significant contribution to future research in minimally invasive dentistry. At the same time, the red and green groups contained the documents that were most closely related, being followed by groups 3, 4 and 5.

**Figure 3:**
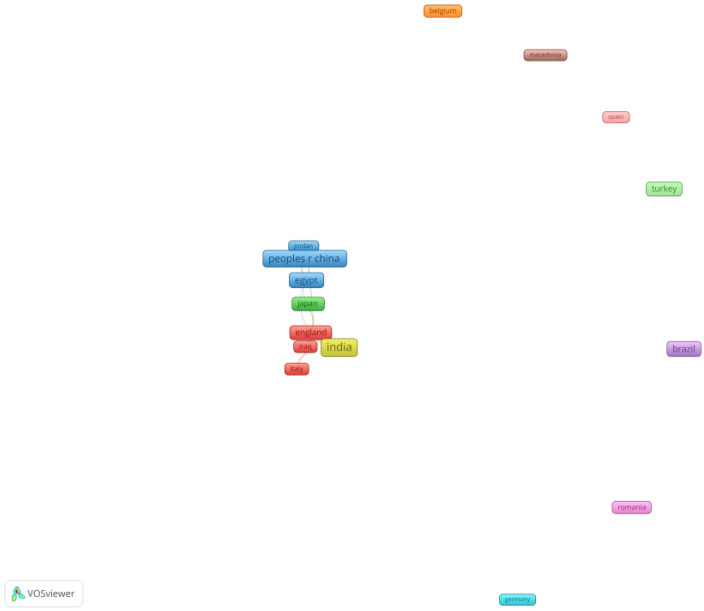
Scientific co-authorship by country. Source: own processing through VOSviewer.

**Figure 4:**
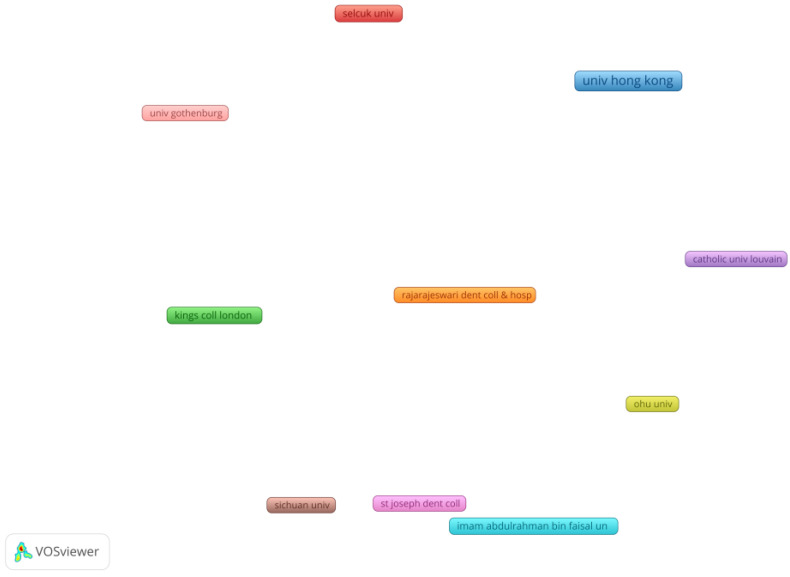
Scientific co-authorship by university. Source: own processing through VOSviewer.

In the last part of the research, we conducted an analysis of the scientific co-authorship and examined the structure of collaboration networks in the field of CMCR methods. The analysis identified the behavior of the research teams, and also their network relationships. The nodes represent either countries or institutions. The degree of collaboration is given by the distance between the nodes. We performed two analyses of scientific co-authorship: by country (Figure 3 and Table 3) and by university (Figure 4 and Table 4).

Figure 3 shows 29 countries in 11 groups. Our results indicate that the geographical area that analyzed the discussed topics was rather large, but we could see strong links between different countries in different parts of the world. The most relevant groups were the first four. They are represented by different colors: red, green, blue and yellow. England was the European country with the most diverse collaboration teams (six documents), and with strong relationships with other countries. Outside Europe, countries such as Australia (eight documents), Egypt (seven documents), the People’s Republic of China (11 documents), India (16 documents) and Saudi Arabia (five documents) maintained a broad range of cooperation with other countries. Authors from Brazil (seven documents), and Turkey (five documents) published several articles on the topic of CMCR methods, but they limited their collaboration among the researchers from their respective countries.

Out of 123 institutions, 17 institutions had a minimum of two published documents. These were grouped in 10 clusters, as shown in Figure 4 and Table 4.

### 3.3. Co-Citation Network

**Figure 5:**
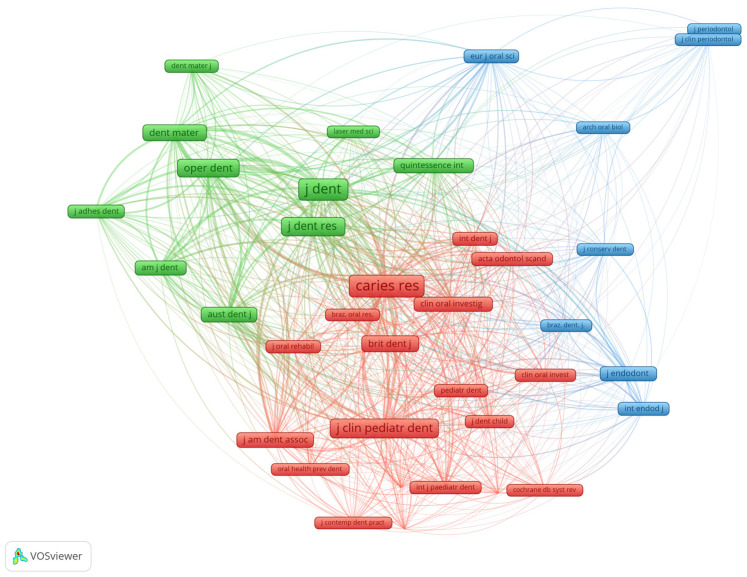
Network of co-citation of scientific journals. Source: own processing through VOSviewer.

This section of the analysis focused on the network of co-citation of scientific sources (scientific journals) in our research area. Regarding the co-citation links, the distance between two nodes (sources) indicates the connection between them. Thus, a small distance between nodes indicates a stronger link, while a large distance between nodes indicates a weaker link. Co-citation of scientific sources shows the frequency with which two sources were cited together by another source. The unit of analysis was represented by the titles of the sources extracted from the raw reference strings in the database. Out of 392 sources, only 37 met the threshold of at least 10 citations per source. Therefore, the analysis highlighted only the most productive journals in our research area, which were divided into three different groups (Figure 5 and Table 5).

The first group (red) included a total of 20 sources. The group contained the most cited source (Caries Res—Caries Research), with 183 citations and a total link strength of 3865. Caries Research covers a broad field of clinical, laboratory and epidemiological studies, emphasizing the advances made in caries prevention, dental erosion, dental fluorosis, and caries patterns in different populations and identifying genes or mutations correlated with caries prevalence and other related dental diseases.

The second group (green) consisted of 10 sources, including the second and third most cited sources. The second most important source was the Journal of Dentistry (J Dent), with 176 citations and a total link strength of 4633. The journal covers a wide range of topics from restorative dentistry to oral health and oral biology. On a larger scale, the journal aspires to influence dental practice at the clinician, research, industry, and policy-maker levels.

The third most relevant journal was the Journal of Clinical Paediatric Dentistry (J Clin Pediatr Dent), with 126 citations and a total link strength of 2999. This journal focuses on the publication of relevant and useful data about the latest innovations in the field of pediatric dentistry. The source gathers information regarding prevention measures and therapeutic management of diverse medical and dental problems related to children.

Operative Dentistry (Oper Dent; 97 citations; 2758 total link strength) and Dental Materials (Dent Mater; 74 citations; 2004 total link strength) were two other significant sources included in the green group.

The third group (blue) included sources from several fields of study (periodontics, endodontics, conservative dentistry, oral biology). The most relevant sources in this group were the Journal of Endodontics (J Endodont; 47 citations; 769 total link strength) and the European Journal of Oral Sciences (Eur J Oral Sci; 34 citations; 931 total link strength).

We can say that, overall, these journals are the most significant sources with the most important contributions in our field of research.

The present study has some limitations. The data used for analysis were limited to original articles and review articles written in English included in WoS Core Collection, and no additional academic research databases were taken into account. The search criteria addressed a limited timeframe from January 2010 to December 2020 and referred only to two types of CMCR agents. The bibliometric analysis using specialized software offered an objective perspective on the topic of interest, but the interpretation of the results could have been somewhat subjective.

The implementation of minimally invasive procedures on a larger scale in the standard therapeutic protocols could be more thoroughly analyzed in future studies by extending data collection to other types of publications (e.g., proceedings papers), other academic research databases, over a longer time frame, and by including more CMCR agents and techniques in the search criteria, which may provide further insight into the latest findings in the research field.

## 4. Conclusions

Our analysis showed a worldwide interest in the subject of chemo-mechanical methods of caries removal, which has transcended the area of pediatric dentistry. These minimally invasive methods were originally intended for use in pediatric patients, but the ongoing epidemiological context has triggered a growing interest in the subject, shining new light upon the use of chemo-mechanical methods of caries removal in adult patients as well, because of their main advantage of not being aerosol generators.

The bibliometric analysis approach reflected the state of knowledge in the studied field and presented the existing links between authors, sources, institutions and countries that are actively involved in research worldwide. Our results highlight the importance of chemo-mechanical agents when compared to conventional treatment options, providing improved patient comfort during dental procedures and contributing to an enhanced level of communication and treatment acceptance. The analyzed studies have been conducted mainly in high-income countries that have developed sanitary policies regarding the prevention and early treatment of carious lesions as a health priority.

## Figures and Tables

**Table 1:** Keyword groups. Source: own processing through VOSviewer.

Word No.	Group 1 (Red)	Occ.	T.L.S.	Group 2 (Green)	Occ.	T.L.S.	Group 3 (Blue)	Occ.	T.L.S.	Group 4 (Yellow)	Occ.	T.L.S.
1	Adhesion	4	26	Carious dentin	21	137	Adhesive systems	13	89	Clinical evaluation	12	76
2	Bond strength	4	29	Cavity preparation	6	43	Caries removal	30	188	Deciduous teeth	7	43
3	Chemo-mechanical caries removal	37	213	Dental pulp	4	19	Carisolv	47	260	Dental caries	19	111
4	Enamel	6	44	Er:YAG laser	9	70	Composite resin	8	57	Dentin	20	101
5	Papacárie	19	103	Fluorescence	5	34	Glass ionomer cement	5	18	Efficacy	14	81
6	Papain	4	26	In vitro	8	53	Interface	4	29	Pain	7	42
7	Self-etch adhesives	8	60	Micro-computerized tomography	5	37	Microtensile bond strength	7	60	Pediatric patients	7	45
8	Smear layer	4	25	Minimally invasive dentistry	9	52	Removal	7	32	Primary teeth	8	61
9	Sodium hypochlorite	6	26	Scanning electron microscopy	6	42	Sound dentin	5	34	Rotary instruments	8	50
10	System	7	48									
11	Teeth	8	44									

Occ., occurrences; T.L.S., total link strength; Er:YAG, erbium-doped yttrium aluminum garnet.

**Table 2:** Author groups and related number of documents and links. Source: own processing through VOSviewer.

Group 1 (Red)	Doc.	Cit.	T.L.S.	Group 2 (Green)	Doc.	Cit.	T.L.S.	Group 3 (Dark Blue)	Doc.	Cit.	T.L.S.
Ali, A. H.	2	10	6	Cebe, M. A.	3	28	9	Coutinho, E.	3	93	9
Banerjee, A.	4	45	10	Karaarslan, E. S.	3	28	9	De Munck, J.	3	93	9
Foschi, F.	2	10	6	Ozturk, B.	2	18	6	Neves, A. A.	3	93	9
Mannocci, F.	4	45	10	Yegin, Z.	2	26	7	Van Meerbeek, B.	3	93	9
Watson, T. F.	2	35	4	Yildiz, E.	2	26	7				
**Group 4 (Yellow)**	**Doc.**	**Cit.**	**T.L.S.**	**Group 5 (Purple)**	**Doc.**	**Cit.**	**T.L.S.**	**Group 6 (Light Blue)**	**Doc.**	**Cit.**	**T.L.S.**
Burrow, M. F.	7	92	16	Hossain, M.	2	5	6	Basting, R. T.	2	29	4
Hamama, H.	7	92	16	Kimura, Y.	2	5	6	Amaral, F. L. B.	2	29	4
King, N. M.	2	27	6	Masuda, Y.	2	5	6	Florio, F. M.	2	29	4
Yiu, C. K. Y.	7	92	16	Yamada, Y.	2	5	6				
**Group 7 (Orange)**	**Doc.**	**Cit.**	**T.L.S.**	**Group 8 (Brown)**	**Doc.**	**Cit.**	**T.L.S.**	**Group 9 (Pink)**	**Doc.**	**Cit.**	**T.L.S.**
Maru, Viral P.	2	6	2	Andrian, S.	2	4	4	Alhumaid, J.	2	2	0
Nagarathna, C.	2	8	3	Pancu, G.	2	4	4				
Shakuntala, B. S.	2	8	3	Stoleriu, S.	2	4	4				
**Group 10 (Light Green)**	**Doc.**	**Cit.**	**T.L.S.**								
Almhoejd, U. S.	2	15	0								

Doc., documents; Cit., citations; T.L.S., total link strength.

**Table 3:** Published documents grouped by country. Source: own processing through VOSviewer.

Group 1	Doc.	Cit.	T.L.S.	Group 2	Doc.	Cit.	T.L.S.	Group 3	Doc.	Cit.	T.L.S.
England	6	55	8	Bangladesh	1	1	1	Australia	8	90	13
Iraq	2	10	3	Czech Republic	1	1	4	Egypt	7	77	14
Italy	2	9	3	Indonesia	1	1	4	Jordan	1	3	1
Norway	1	8	1	Japan	4	13	6	People’s Republic of China	11	122	12
Portugal	1	2	2	Mexico	2	7	1				
Russia	2	2	4								
Sweden	2	15	2								
**Group 4**	**Doc.**	**Cit.**	**T.L.S.**	**Group 5**	**Doc.**	**Cit.**	**T.L.S.**	**Group 6**	**Doc.**	**Cit.**	**T.L.S.**
India	16	66	3	Brazil	7	40	1	Germany	1	31	1
Malaysia	1	0	1	Syria	1	2	1	Taiwan	1	31	1
Saudi Arabia	5	16	3	USA	2	2	2				
**Group 7**	**Doc.**	**Cit.**	**T.L.S.**	**Group 8**	**Doc.**	**Cit.**	**T.L.S.**	**Group 9**	**Doc.**	**Cit.**	**T.L.S.**
Belgium	3	93	0	Macedonia	1	0	0	Romania	3	8	0
**Group 10**	**Doc.**	**Cit.**	**T.L.S.**	**Group 11**	**Doc.**	**Cit.**	**T.L.S.**				
Spain	1	8	0	Turkey	5	31	0				

Doc., documents; Cit., citations; T.L.S., total link strength.

**Table 4:** Published documents grouped by university. Source: own processing through VOSviewer.

Group 1	Doc.	Cit.	T.L.S.	Group 2	Doc.	Cit.	T.L.S.	Group 3	Doc.	Cit.	T.L.S.
Abant Izzet Baysal Univ.	2	12	3	I.M. Sechenov First Moscow State Med. Univ.	2	2	1	Mansoura Univ.	5	63	10
Gaziantep Univ.	2	26	3	Kings Coll. London	3	20	1	Univ. Hong Kong	6	81	10
Selcuk Univ.	3	28	4	Univ. Baghdad	2	10	2	Univ. Melbourne	6	66	10
**Group 4**	**Doc.**	**Cit.**	**T.L.S.**	**Group 5**	**Doc.**	**Cit.**	**T.L.S.**	**Group 6**	**Doc.**	**Cit.**	**T.L.S.**
Ohu Univ.	2	5	2	Catholic Univ. Louvain	2	69	0	Imam Abdulrahman Bin Faisal Univ.	3	4	0
Showa Univ.	2	5	2								
**Group 7**	**Doc.**	**Cit.**	**T.L.S.**	**Group 8**	**Doc.**	**Cit.**	**T.L.S.**	**Group 9**	**Doc.**	**Cit.**	**T.L.S.**
RajaRajeswari Dent. Coll. & Hosp.	2	8	0	Sichuan Univ.	2	14	0	St. Joseph Dent. Coll.	2	9	0
**Group 10**	**Doc.**	**Cit.**	**T.L.S.**								
Univ. Gothenburg	2	15	0								

Doc., documents; Cit., citations; T.L.S., total link strength.

**Table 5:** Citation groups and related link strength in scientific journals. Source: own processing through VOSviewer.

Group 1 (Red)	Cit.	T.L.S.	Group 2 (Green)	Cit.	T.L.S.	Group 3 (Blue)	Cit.	T.L.S.
Acta Odontol. Scand.	30	771	Am. J. Dent.	45	1224	Arch. Oral Biol.	10	223
Braz. J. Oral Sci.	11	358	Aust. Dent. J.	63	1705	Braz. Dent. J.	17	486
Braz. Oral Res.	15	417	Dent. Mater.	74	2004	Eur. J. Oral Sci.	34	931
Brit. Dent. J.	67	1655	Dent. Mater. J.	21	635	Int. Endod. J.	32	627
Caries Res.	183	3865	J. Adhes. Dent.	36	1075	J. Clin. Periodontol.	17	184
Clin. Oral Invest.	21	547	J. Dent.	176	4633	J. Conserv. Dent.	17	335
Clin. Oral Investig.	41	1097	J. Dent. Res.	108	2842	J. Endodont.	47	769
Cochrane Db. Syst. Rev.	11	301	Laser Med. Sci.	15	406	J. Periodontol.	14	134
Indian J. Dent. Res.	15	346	Oper. Dent.	97	2758			
Int. Dent J.	31	866	Quintessence Int.	45	1205			
Int. J. Paediatr. Dent.	26	665						
J. Am. Dent. Assoc.	58	1526						
J. Clin. Pediatr. Dent.	126	2999						
J. Contemp. Dent. Pract.	13	370						
J. Dent. Child.	27	681						
J. Indian Soc. Pedod. Prev. Dent.	19	466						
J. Oral Rehabil.	27	720						
Oral Health Prev. Dent.	16	355						
Pediatr. Dent.	27	691						

Cit., citations; T.L.S., total link strength.

## Data Availability

Additional data supporting the reported results can be requested from the corresponding authors.
